# Age-related differences in the neural bases of phonological and semantic processes in the context of task-irrelevant information

**DOI:** 10.3758/s13415-018-00671-2

**Published:** 2018-11-28

**Authors:** Michele T. Diaz, Micah A. Johnson, Deborah M. Burke, Trong-Kha Truong, David J. Madden

**Affiliations:** 1 Department of Psychology, Pennsylvania State University, University Park, PA 16803 USA; 2 Department of Psychology, University of California, Los Angeles, Los Angeles, CA USA; 3 Brain Imaging and Analysis Center, Duke University School of Medicine, Durham, NC USA; 4 Department of Linguistics and Cognitive Science, Pomona College, Claremont, CA USA; 5 Department of Psychiatry and Behavioral Sciences, Duke University School of Medicine, Durham, NC USA

**Keywords:** Aging, fMRI, Semantics, Phonology, Language production

## Abstract

As we age we have increasing difficulty with phonological aspects of language production. Yet semantic processes are largely stable across the life span. This suggests a fundamental difference in the cognitive and potentially neural architecture supporting these systems. Moreover, language processes such as these interact with other cognitive processes that also show age-related decline, such as executive function and inhibition. The present study examined phonological and semantic processes in the presence of task-irrelevant information to examine the influence of such material on language production. Older and younger adults made phonological and semantic decisions about pictures in the presence of either phonologically or semantically related words, which were unrelated to the task. FMRI activation during the semantic condition showed that all adults engaged typical left-hemisphere language regions, and that this activation was positively correlated with efficiency across all adults. In contrast, the phonological condition elicited activation in bilateral precuneus and cingulate, with no clear brain–behavior relationship. Similarly, older adults exhibited greater activation than younger adults in several regions that were unrelated to behavioral performance. Our results suggest that as we age, brain–behavior relations decline, and there is an increased reliance on both language-specific and domain-general brain regions that are seen most prominently during phonological processing. In contrast, the core semantic system continues to be engaged throughout the life span, even in the presence of task-irrelevant information.

**Electronic supplementary material:**

The online version of this article (10.3758/s13415-018-00671-2) contains supplementary material, which is available to authorized users.

In language production, one of the most commonly reported challenges for older adults is word retrieval (for a review, see Burke & Shafto, 2008). These retrieval deficits are hypothesized to arise, at least in part, from impaired phonological retrieval (Burke, Locantore, Austin, & Chae, 2004; James & Burke, 2000; MacKay & James, 2004; Maylor, 1990; Vitevitch & Sommers, 2003). Consistent with this hypothesis, older adults report an increased number of tip of the tongue experiences, where one knows the meaning of a word but is unable to produce the phonology (Brown & McNeill, 1966; Burke, Mackay, Worthley, & Wade, 1991). In contrast, older adults tend to show comparable performance, relative to younger adults, in most forms of semantic retrieval. Relative to younger adults, older adults have similar levels of semantic priming (Bowles, Williams, & Poon, 1983; Burke, White, & Diaz, 1987; Madden, Pierce, & Allen, 1993), stable or increased vocabulary scores (Kemper & Sumner, 2001; Singer, Verhaeghen, Ghisletta, Lindenberger, & Baltes, 2003; Verhaeghen, 2003), and more lexically diverse language production (Kemper & Sumner, 2001), at least until their 70s (Barresi, Nicholas, Connor, Obler, & Albert, 2000; Verhaegen & Poncelet, 2013). These observations point to a larger and more elaborate semantic system for older adults, although there is considerable individual variability in semantic processing, particularly at older ages, that may be influenced by educational quality (Paolieri, Marful, Morales, & Bajo, 2018), cognitive ability (Federmeier, McLennan, De Ochoa, & Kutas, 2002; Singer et al., 2003), and habits (Payne, Gao, Noh, Anderson, & Stine-Morrow, 2012). Moreover, these contrasting patterns of decline and retention in phonology and semantics suggest that these aspects of language have different cognitive and neural bases. Indeed, the transmission deficit theory proposes that while all connections decline with age, the phonological system is particularly vulnerable to decline because it has fewer converging connections (Burke, MacKay, & James, 2000; Burke et al., 1991). On the other hand, because the semantic system is more heavily interconnected, weaker links are less likely to produce a behavioral deficit.

Phonological and semantic processes, however, interact with other aspects of cognition, such as working memory and executive function, which tend to show age-related decline. Inhibition, one component of executive function, refers to the ability to ignore or suppress prepotent responses or irrelevant information (Miyake et al., 2000). Indeed, during language production in healthy younger adults, there is strong evidence that overt production involves inhibition (Shao, Meyer, & Roelofs, 2013; Shao, Roelofs, Martin, & Meyer, 2015; Shao, Roelofs, & Meyer, 2012), likely at the level of response selection (Piai, Roelofs, & Schriefers, 2014). Hasher and Zacks (1988) proposed that age-related declines in the ability to inhibit irrelevant information leads to age-related slowing. For example, in language comprehension, older adults are slowed more than younger adults are when text is interspersed with distracting text, particularly when the distracting text is related to the target text (Connelly, Hasher, & Zacks, 1991), or when it is presented in an unpredictable location (Carlson, Hasher, Zacks, & Connelly, 1995). Other behavioral studies examining interactions between executive function and language production have shown that under dual task conditions, older adults are able to modify their rate of speech without compromising other factors such as fluency and grammatical complexity (Kemper, Herman, & Lian, 2003; Kemper, Schmalzried, Herman, Leedhal, & Mohankumar, 2009), at least until task demands become very high (Kemper, Schmalzried, Hoffman, & Herman, 2010). Others have shown that variability in verbal fluency, a combined measure of both production and executive function, is related to the use of prediction during language comprehension (Federmeier et al., 2002), again demonstrating the interaction of domain-general resources with both language production and comprehension. Beyond age-related declines in inhibition and executive function, older adults may be slower in suppressing early sensory aspects of such distracting information (Clapp & Gazzaley, 2012; Gazzaley et al., 2008), and may have difficulty switching between functional networks (Clapp, Rubens, Sabharwal, & Gazzaley, 2011) or maintaining connectivity between frontal and sensory regions (Clapp, Rubens, & Gazzaley, 2010; Solesio-Jofre et al., 2011).

Neuroimaging techniques can be used to examine the neural bases of age-related differences in language, inhibition, and their interaction. Both younger and older adults rely on a core set of regions to support language comprehension and production, including the left inferior frontal gyrus, left supramarginal and angular gyri, as well as bilateral and lateral temporal cortices (Hickok & Poeppel, 2004; Indefrey & Levelt, 2004; Silbert, Honey, Simony, Poeppel, & Hasson, 2014). Neuroimaging studies suggest inhibition and conflict monitoring during overt production rely on regions outside of this core language network, such as the anterior cingulate (Abutalebi & Green, 2007; Piai, Roelofs, Acheson, & Takashima, 2013a) as well as the right lateral prefrontal cortex (Fedorenko, Duncan, & Kanwisher, 2012; Roelofs, 2008), and anterior regions such as these are known to decline with age (Raz, Gunning-Dixon, Head, Dupuis, & Acker, 1998; Resnick, Pham, Kraut, Zonderman, & Davatzikos, 2003; Salat et al., 2004). Age-related increases in fMRI activation are commonly found. However, there is disagreement about whether age-related differences are compensatory (e.g., Reuter-Lorenz & Cappell, 2008), represent neural dedifferentiation (e.g., Li, Lindenberger, & Sikstrom, 2001), or reflect a combination of both (Martins, Joanette, & Monchi, 2015; Reuter-Lorenz & Cappell, 2008). Indeed, some have suggested that older adults experience temporal delays in neural engagement, particularly of prefrontal regions that underlie executive function. These delays may underlie some of the complex age-related differences that have been observed, where small delays in relatively simple tasks do not affect performance, but delays in complex tasks lead to more substantial impairments in performance (Martins et al., 2015).

The earliest reports examining picture naming largely supported a compensatory pattern, showing that older adults demonstrated similar accuracy compared with younger adults while eliciting increased activation in the right inferior frontal gyrus and bilateral cingulate (Wierenga et al., 2008). Moreover, older adults’ increased activation in the right inferior frontal gyrus was positively correlated with accuracy. Similarly, Shafto and colleagues reported that older adults’ increased activation in the insula during word retrieval was associated with fewer tip-of-the-tongue states (Shafto, Stamatakis, Tam, & Tyler, 2010). Effects consistent with compensation have also been reported during a lexical rhyme judgment task (Geva et al., 2012), in which activation in the right inferior frontal gyrus (IFG) increased with age, while older adults responded with similar speed to younger adults. However, in that same study, older adults who had the highest error rates elicited the largest right IFG activation, suggesting that increased activation may reflect compensatory effort, but not necessarily successful performance. Indeed, individual performance can vary, and variability is typically greater among older adults. Interestingly, a split-half analysis of higher and lower performing older adults in the Wierenga et al. (2008) picture-naming study showed opposite patterns: High performers had a positive correlation between accuracy and rIFG fMRI activation, while lower performers had a negative relation between these variables (Wierenga et al., 2008). These findings suggest that there are complex relations between age-related differences in activation and behavioral performance, and that more than one neurocognitive profile may exist.

Previous research from our lab comparing phonological and semantic decisions has shown that older adults were less accurate and less efficient in making phonological judgments about pictures, whereas performance did not differ for semantic judgments (Diaz, Johnson, Burke, & Madden, 2014). Analyses of the fMRI activation related to these decisions showed that while the semantic task engaged typical left hemisphere language regions, the phonological task engaged bilateral cingulate and precuneus, suggesting a greater reliance on task-control regions during the phonological task. Consistent with previous findings, older adults exhibited greater activation than younger adults did throughout the brain. However, these increases in activation were not related to behavioral performance for older adults, consistent with a dedifferentiation account. Other work by our group has examined the interaction of executive processes and language using lexical competition. In a picture-word interference task, during the categorical condition, we found that older adults engaged the left middle frontal gyrus more than younger adults did, suggesting a greater need for domain-general inhibitory mechanisms when interference demands are high (Rizio, Moyer, & Diaz, 2017). Similarly, in a semantic judgment task, we found that older adults relied more heavily on the left inferior frontal gyrus and right posterior cingulate when semantic competition was high (Zhuang, Johnson, Madden, Burke, & Diaz, 2016). These findings suggest that older adults rely more heavily on domain-general inhibitory and monitoring regions, particularly when task demands are high.

The goals of the present study were to extend our previous findings from Diaz et al. (2014), in which we found that older adults were less efficient than younger adults when making phonological, but not semantic judgments. Our primary goal of the present study is to examine the influence of additional, task-irrelevant information on phonological and semantic judgments in younger and older adults to examine potential age-related inhibitory and/or phonological differences. On the basis of these previous findings, we predicted that if older adults are less efficient than younger adults in the inhibition of irrelevant information, then age-related differences in behavior and activation should occur across both conditions. Specifically, if older adults’ differences are inhibitory based, then we would expect older adults to be slower and less accurate on both conditions, and to show increased activation compared with younger adults in domain-general regions associated with executive function and task control, such as the middle frontal gyrus and anterior cingulate. However, if older adults have a greater age-related impairment in phonological processing, then we would expect to see an interaction effect, such that larger age-related differences are found for the phonological condition, with age-related differences in regions that correspond to phonological processing, such as the left posterior, inferior frontal gyrus, left precentral gyrus, and left supramarginal gyrus. To better understand these neural mechanisms, we examined the relations between fMRI activation and behavior, and how these relationships may differ across age groups and conditions. Specifically, we examined the results for patterns of neural compensation or inefficiency in older adults. Increases in activation for older adults that correspond to maintained or improved behavior would be consistent with a compensation account. Whereas weaker or no relation between increased fMRI activation and behavioral performance in older adults would be consistent with neural inefficiency. Finally, we compared the results from the present study to Diaz et al. (2014) to evaluate the effect of task-irrelevant information on phonological and semantic processing.

## Method

### Participants

All participants were community-dwelling, right-handed, native English speakers (20 younger adults: *M*_age_ 25.0, age range: 19–35 years, eight male; and 20 older adults: *M*_age_ 67.5 years, age range: 59–76 years, eight male). One younger participant was removed for a high number of omitted responses during the task, and one older participant was removed due to anxiety in the scanner, leaving 19 participants in each group. All participants reported normal or corrected-to-normal vision. No one was color-blind, and none reported a history of neurological disorders, psychological disorders, or major medical conditions (e.g., diabetes, heart disease; Christensen, Moye, Armson, & Kern, 1992). All participants completed neuropsychological testing to assess basic cognitive skills such as speed, memory, executive function, and language. Across groups, participants did not differ in handedness, years of education, Mini-Mental State Exam (MMSE) scores, measures of anxiety and depression, vocabulary, verbal fluency, digit-symbol forward, or recall. Demographic characteristics are presented in Table 1. Each participant provided informed consent and was paid for his or her participation. All experimental procedures were approved by the Duke University School of Medicine Institutional Review Board, where the study was conducted.

**Table 1:** Participant demographics

	Younger	Older
N	19	19
Age***	25.0 (4.90)	67.47 (5.36)
Handedness	77.65 (24.98)	80.57 (23.13)
Education	16.37 (2.14)	17.16 (2.01)
MMSE	29.16 (1.01)	29.26 (0.81)
HADS	0.05 (0.08)	0.07 (0.08)
Vocabulary (WAIS II)	57.63 (5.74)	57.37 (4.94)
Verbal fluency (total)	72.32 (18.45)	66.37 (16.57)
Digit symbol (DS) RT***	1271.48 (239.16)	1861.49 (350.84)
DS forward	12.63 (2.71)	11.42 (2.39)
DS backward**	9.63 (2.67)	7.68 (1.83)
Stroop RT***	492.42 (88.54)	647.40 (167.56)
Speed RT***	294.87 (40.07)	356.25 (64.02)
Immediate recall	11.68 (3.04)	10.79 (2.74)
Delayed recall	10.89 (3.65)	9.00 (3.32)

Values provided are means and (standard deviations). **p < .01. ***p < .0001. MMSE = Mini-Mental State Exam; HADS = Hospital Anxiety and Depression Scale; WAIS = Wechsler Adult Intelligence Scale; RT = reaction time

### Experimental task

**Fig. 1:**
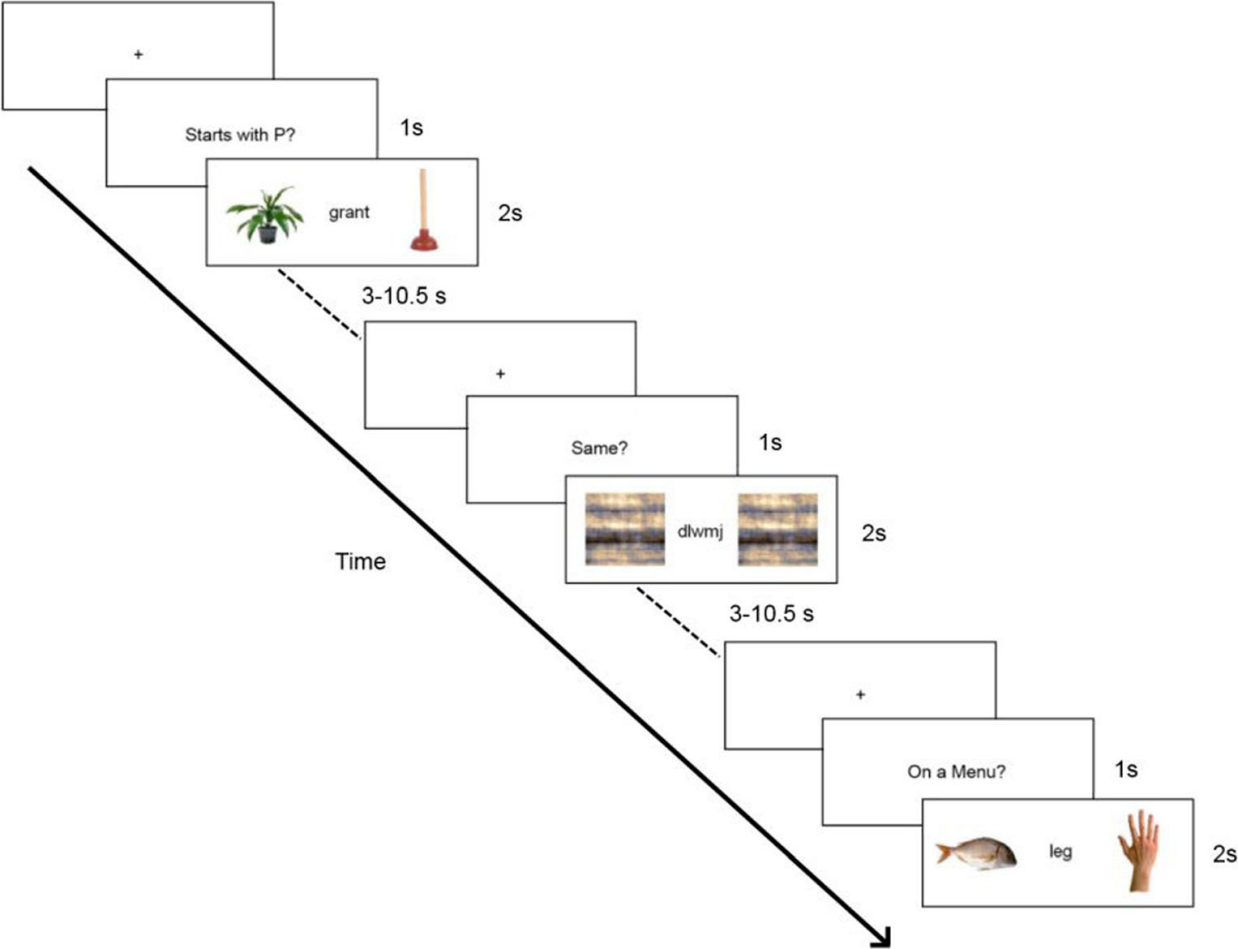
Overview of task design. Typical trials (phonological match, perceptual match, and semantic nonmatch). Cues were presented for 1 s, followed by pictures and additional task-irrelevant words that were presented for 2 s. The interstimulus interval was jittered between 3 and 10.5 s with a mean interval of 4.8 s

Figure 1 provides an overview of the task design. The main objective of the present experiment was to examine the influence of additional, task-irrelevant information on semantic and phonological processing in order to better understand competing theoretical accounts that posit age-related decline in inhibitory or phonological processes. Each trial consisted of a cue (phonological, semantic, or perceptual, duration = 1 s), followed by two photographs presented side by side (duration = 2 s). Simultaneously presented with the photographs was a written word that was phonologically, semantically, or perceptually related to the photographs. Participants were asked to decide if both photographs matched the cue or not and made their response manually with the index and middle fingers of the right hand. They were instructed to respond as quickly as possible while maintaining accuracy.

### Stimuli

Cues were a word or short statement followed by a question mark. Phonological cues presented a question about the first letter of the object names (e.g., Starts with *B*?, Starts with *P*?).^1^ Semantic cues presented a question about a functional or perceptual feature of the objects (e.g., Smooth?, Flies?, Edible?). Perceptual cues probed if the two items were identical (e.g., Same?). For the phonological and semantic conditions, the target stimuli were photographs of everyday objects. For the perceptual condition, photographs were phase-transformed versions of a subset of the object photographs that retained the basic perceptual features of the photographs, but did not form a recognizable object (examples of the original and perceptual images can be found in Fig. S1 in the Supplemental Materials). For the phonological and semantic conditions, the names of the objects did not statistically differ in length (number of letters; phonological = 5.53; semantic = 5.55), number of syllables (phonological = 1.62; semantic = 1.64), number of phonemes (phonological = 4.77; semantic = 4.61), or word frequency (SUBTLEX corpus; Brysbaert & New, 2009; phonological = 34.76; semantic = 33.29). Ratings were obtained from the English Lexicon Project (Balota et al., 2007). All stimuli were selected based on two separate behavioral pretests, as previously reported (Diaz et al., 2014), and items were counterbalanced between the phonological and semantic conditions across participants with two separate lists of items.

The relationship of the additional, task-irrelevant word to the photographs varied by condition. On phonological trials, the word rhymed with one of the photographs. On semantic trials, the word was semantically related to at least one of the photographs, but for some semantic-match trials, it related to both items. These additional task-irrelevant words did not statistically differ across conditions in length or word frequency. Perceptual information consisted of random consonant strings. In this way, the additional words were always related to one of the objects (phonologically, semantically, or perceptually), but did not convey information that facilitated the task decision.

### Acquisition of MRI data

MRI scanning was completed on a 3.0 Tesla GE MR 750 whole-body 60 cm bore human scanner equipped with 50 mT/m gradients and a 200 T/m/s slew rate. An eight-channel head coil was used for radio frequency reception (General Electric, Milwaukee, WI, USA). Sagittal T-1 weighted localizer images were acquired and used to define a volume for data collection and high-order shimming. The anterior and posterior commissures were identified for slice selection and shimming. A semiautomated high-order shimming program was used to ensure global field homogeneity. High-resolution structural images were acquired using a 3-D fSPGR pulse sequence (TR = 8.14 ms; TE = 3.22 ms; TI = 450 ms; FOV = 24 cm^2^; flip angle = 12°; voxel size = 0.9375 × 0.9375 × 1mm; matrix = 256 × 256; 162 contiguous slices; averages = 1; phase encoding: RL; bandwidth: 62.5 kHz). Functional images sensitive to blood-oxygen-level-dependent (BOLD) contrast were acquired using an inverse spiral pulse sequence with SENSE acceleration (TR = 2.0s; TE = 30 ms; FOV = 25.6 cm^2^; flip angle = 60°; SENSE factor = 2; voxel size = 3.75 × 3.75 × 4 mm; matrix = 64 × 64; 38 contiguous oblique axial slices, parallel to the AC-PC line, interleaved acquisition; bandwidth: 250 kHz). Four initial RF excitations were performed to achieve steady state equilibrium and were subsequently discarded.

The entire experiment comprised 240 trials, with 80 trials (40 match, 40 nonmatch) in each of the three judgment conditions. Trial types were presented in a randomized order across eight, 4-minute fMRI runs, with a variable intertrial interval (ITI; interval = 3–10.5 s, *M* = 4.8 s). Trial order across conditions and ITIs were randomized and optimized using the Optseq2 program (Dale, 1999). Each run began and ended with the presentation of a fixation cross, 6 s and 15 s, respectively, and a fixation cross was also presented during the variable interval between each trial. All stimuli were presented via a projector using an in-house experimental control program (Voyvodic, 1999). Responses were recorded with a hand-held fiber optic response box (Current Designs, Philadelphia, PA, USA).

### FMRI data analysis

Data were analyzed for quality via a tool that quantifies several metrics including signal-to-noise (SNR), signal-fluctuation-to-noise (SFNR), motion, and voxel-wise standard deviation measurements (Friedman & Glover, 2006; Glover et al., 2012). Additionally, all data were visually inspected for artifacts and blurring. The average movement in the *X, Y,* or *Z* directions was .23 mm (range: .04–1.25 mm). Thus, none of the included participants moved more than one half voxel in the *X, Y,* or *Z* dimensions. We used FSL Version 5.0.1 and FEAT Version 6.00 for preprocessing and for all analyses of functional activations (Smith et al., 2004; Woolrich et al., 2009). Prewhitening or voxel-wise temporal autocorrelation was estimated and corrected using FMRIB’s Improved Linear Model (FILM; Woolrich, Ripley, Brady, & Smith, 2001). The skull and other coverings were stripped from the structural brain images using the FSL brain extraction tool (Smith, 2002). Functional image data were corrected for slice timing using sinc interpolation to shift each slice in time to the middle of the TR period. Functional images were motion-corrected using FSL’s MC-FLIRT (FMRIB's Linear Image Registration Tool) using six rigid-body transformations (Jenkinson, Bannister, Brady, & Smith, 2002). These estimates of motion were included as nuisance covariates in the overall FSL model. Functional data were also high-pass filtered (cutoff = 50 s), and spatially smoothed using a Gaussian kernel (FWHM = 8 mm). Functional images of each participant were coregistered to the participant’s structural images in native space, and structural images were normalized to Montreal Neurological Institute (MNI) standard space using FSL’s MNI Avg152, T1 2 × 2 × 2 mm standard brain. The same transformation matrices used for structural-to-standard transformations were then used for functional-to-standard space transformations of coregistered functional images. Coregistration and normalization steps were completed using a combination of affine and nonlinear registrations (Greve & Fischl, 2009; Jenkinson et al., 2002; Jenkinson & Smith, 2001).

We used a double γ function to model the hemodynamic response for each trial, time-locked to the onset of the pictures, although this likely included some overlap from processing the cue as well. Correct trials were modeled by condition, and errors (incorrect responses and omitted responses) were modeled as a separate regressor. Reaction time (RT) outliers were defined as responses < 250 ms or + 3 standard deviations from that individual's overall mean. Collectively, these accounted for approximately 9% of the trials (7.2% incorrect, 0.47% no response, 0.95% outliers).

We combined the analyses from each experimental run and performed an analysis across runs for each participant individually. We then combined these analyses across participants into a group-level analyses using the FMRIB Local Analysis of Mixed Effects (FLAME 1 & 2) to identify voxels that were activated by each condition (Beckmann, Jenkinson, & Smith, 2003; Woolrich, Behrens, Beckman, Jenkinson, & Smith, 2004). Our primary analytic goals were to identify regions that were responsive to our experimental manipulation (phonological and semantic processing), to identify regions where older and younger adults differed, and to detect regions in which the activation differed as a function of both age group and task condition concurrently (i.e., interaction). To accomplish this, we performed a two-way analysis of variance (ANOVA) within FSL with condition (phonological, semantic), age group (younger, older), and the interaction of Condition × Age Group as independent variables. Because perceptual trials mainly served as a control for motor and perceptual activation and were qualitatively different from the language conditions, they were omitted from this ANOVA. Within FSL, we also made comparisons between conditions to identify differences in functional activation between levels of each variable (e.g., phonological > semantic, semantic > phonological). All significant activations were determined using a two-step process in which voxels, significant at *p* < .01, were identified. Clusters of identified voxels were then corrected for multiple comparisons according to Gaussian random fields (GRF) theory (*p* < .05, corrected) in which each cluster's estimated significance level was compared with the cluster probability threshold, and then only clusters whose estimated significance exceeded the threshold were included in the results (Hayasaka & Nichols, 2003). Results from comparisons between conditions (e.g., phonological > semantic) were masked by results from more basic analyses (e.g., significant activation to phonological > perceptual) in a conjunction analysis to limit the comparisons to positive hemodynamic response differences. All analyses involved a whole-brain approach with single comparisons, and thus the comparisons should not be statistically biased (Kriegeskorte, Simmons, Bellgowan, & Baker, 2009). We determined anatomical gyri corresponding to the peaks of activation through reference to anatomical atlases (Desikan et al., 2006). All coordinates are reported in MNI space.

To investigate the relationships between activation and behavior, we conducted three linear regressions in which age group, the parameter estimates of the local maxima of fMRI activation, and the interaction of age group and fMRI activation were independent variables (predictors), and efficiency (i.e., RT/accuracy) was the outcome variable. Local maxima of parameter estimates were collapsed across regions for each group and condition. One regression model was conducted for each of the phonological, semantic, and age effects. Similar analyses conducted with RT yielded similar results and are described in the Supplementary Materials.

## Results

### Behavioral results

**Fig. 2:**
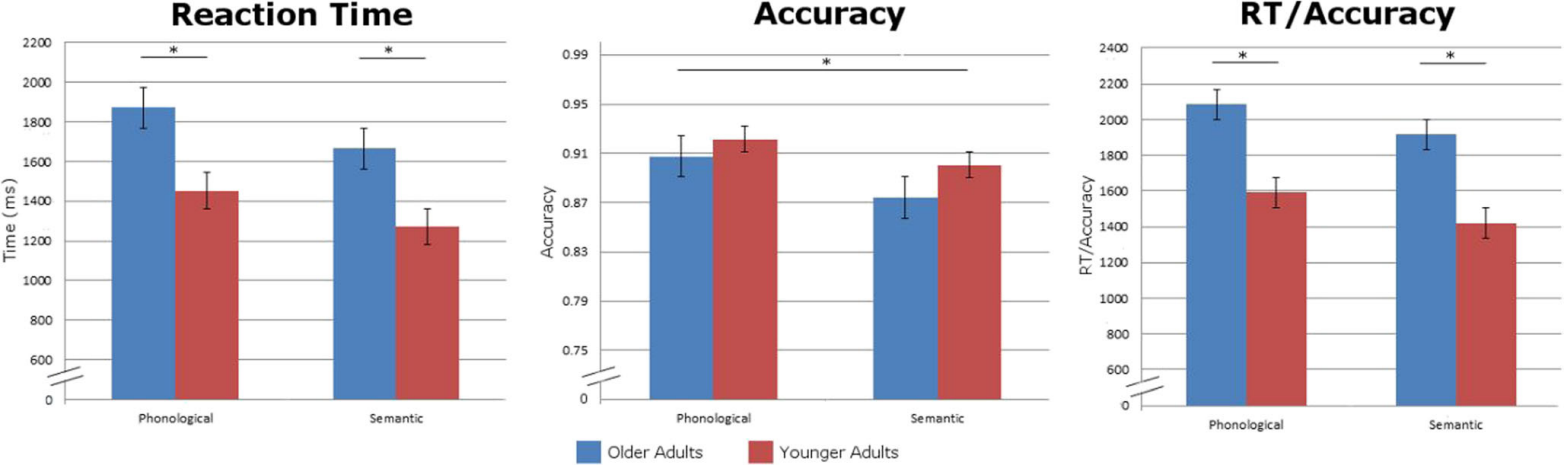
Behavioral results. Group means for reaction time (RT), accuracy, and RT/accuracy with standard error bars. There was a main effect of condition in all three behavioral measures with participants responding more slowly and more accurately to the phonological trials and responding faster but less accurately to the semantic trials. There was also a main effect of age group in the RT and RT/accuracy measures, with older adults responding more slowly overall. There were no significant interactions between age group and condition

Behavioral data are presented in Fig. 2. For all of our behavioral analyses, we performed univariate analyses of variance (ANOVAs) with age group as a between-subjects variable and condition (phonological, semantic) as within-subjects variables. Effects of match type (match, nonmatch) are described in the Supplemental Materials. Because the perceptual task was qualitatively different from the other two conditions and served primarily as a control for baseline fMRI activation of visual and motor cortices, we did not include it in our primary analyses of the behavioral results reported here, but these results can be found in the Supplemental Materials.

#### RT/accuracy

Because there appeared to be speed–accuracy trade-offs that differed as a function of condition (i.e., slower but more accurate performance in the phonological condition relative to the semantic condition), we conducted an analysis of RT divided by accuracy, often referred to as inverse efficiency. Essentially, this analytic approach adjusts RT to incorporate accuracy and can serve as a measure of overall processing efficiency, with higher values representing less efficient performance, similar to RT (e.g., Horowitz & Wolfe, 2003; Townsend & Ashby, 1983). This analysis indicated that efficiency was worse for older adults than for younger adults, *F*(1, 36) = 15.22, *p* < .0005, and was worse in the phonological condition than in the semantic condition, *F*(1, 36) = 30.5, *p* < .0001. Analyses of untransformed RT show largely similar results and are reported in the Supplemental Materials.

#### Accuracy

A mixed-logistic regression was conducted on the number of response errors to explore the effect of condition and age, with random effects of subject and item slopes (Jaeger, 2008). This analysis indicated that there was a marginally significant effect of condition (β = −.36, *SE* = .18, *z* = −1.94, *p* = .052), where participants were slightly more accurate in the phonological condition compared with the semantic condition. There was no significant main effect of age group or interaction between condition and age group.

#### Comparisons with our previous experiment

To further assess the influence of the additional task-irrelevant words on behavioral performance, we conducted a comparison between the behavioral results from the present experiment and those from our earlier experiment in which identical trials were presented without additional words (Diaz et al., 2014). First, we compared the two groups’ cognitive and demographic profiles to assess the comparability of the individuals across experiments. There were no statistically significant differences between the two experiments in education, MMSE, depression ratings, verbal fluency, digit symbol RT, Stroop RT, speed RT, delayed recall, or immediate recall. There was a significant main effect of experiment for vocabulary in which participants from the first experiment had higher vocabulary scores, *F*(3, 66) = 6.84, *p* < .001. There was also a significant Experiment × Group interaction in which younger adults had lower vocabulary scores than older adults in the first experiment only (Exp. 1 younger = 55.00, Exp. 1 older = 62.75, Exp. 2 younger = 57.63, Exp. 2 older = 57.37)*.* Thus, with the exception of small differences in vocabulary, the participants across the two experiments were comparable.

In looking at the task-related behavioral data, there were no significant main effects of experiment for RT/ACC, RT, or accuracy. However, for accuracy there was a marginally significant Experiment × Group × Condition interaction, with older adults from the present experiment performing more accurately on phonological trials than older adults from the first experiment, which did not have additional, task-irrelevant information, *F*(1, 126) = 3.48, *p* = .065. There were no differences in accuracy for the semantic condition between the two studies or comparing older and younger adults.

### FMRI results

#### Results to individual conditions

**Fig. 3:**
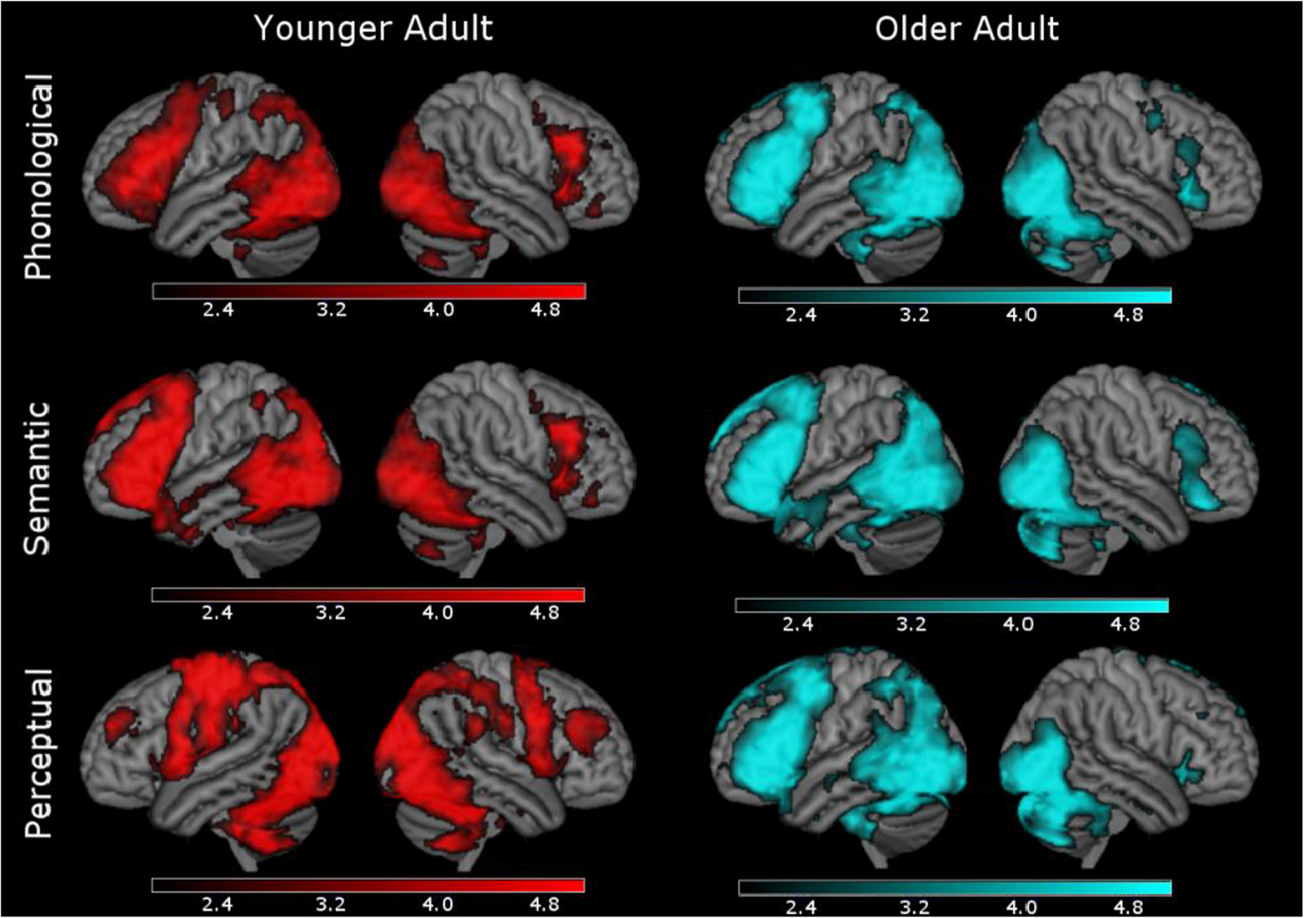
FMRI activations to individual conditions. An overview of the regions in which there was significant activation comparing phonological trials > perceptual control trials, semantic trials > perceptual control trials, and the perceptual control trials > null events for younger and older adults. Light shaded regions represent areas where significant differences were found, *p* < .01 corrected

First, we report the activation to individual conditions compared with our perceptual control condition and activation to our perceptual control condition compared with the overall baseline (see Fig. 3, Table 2). Across participants, the phonological and semantic conditions elicited greater activation than our perceptual control condition did bilaterally in typical language regions, including the bilateral inferior frontal and temporal gyri, as well as cognitive control regions such as the superior and middle frontal gyri and cingulate and paracingulate gyri. Activation was also observed in the bilateral visual cortex and left thalamus. Consistent with previous studies of language, activation was more extensive in the left hemisphere, and for the semantic condition activation was more extensive in temporal cortex. See Table 2 for full details.

**Table 2:** Activations to conditions

Region	Hemisphere	Max *Z*	Peak (MNI)		
			*X*	*Y*	*Z*
Semantic > perceptual					
Orbital frontal cortex	Left	8.66	−38	34	−10
	Right	7.14	48	32	−14
Insula	Right	6.89	34	24	−2
Middle frontal gyrus	Right	5.83	56	26	30
IFG, pars triangularis	Left	9.68	−50	30	14
IFG, pars opercularis	Right	6.51	44	18	24
Paracingulate gyrus	Left	9.20	−4	18	50
Superior frontal gyrus	Left	8.93	−4	12	54
Thalamus	Left	6.24	−8	−14	4
Inferior temporal gyrus	Left	8.66	−44	−56	−8
Lateral occipital cortex	Left	8.25	−40	−78	−4
	Right	9.34	42	−78	−8
Phonological > perceptual					
IFG, pars triangularis	Left	9.39	−44	26	14
Orbital frontal cortex	Right	7.56	32	28	0
IFG, pars opercularis	Left	9.34	−44	6	26
Paracingulate gyrus	Left	8.97	−6	16	48
Cingulate gyrus	Left	6.37	−6	4	28
	Right	6.01	6	4	28
Thalamus	Left	7.81	−12	−14	−10
Occipital fusiform gyrus	Right	9.30	−42	−66	−14
Lateral occipital cortex	Left	9.29	−42	−72	−12
	Right	9.69	40	−80	−8
Perceptual					
Supplementary motor cortex	Left	9.43	−6	6	44
Precentral gyrus	Left	7.58	−54	4	34
Thalamus	Left	8.01	−14	−20	2
Temporo-occipital FFG	Left	9.74	−28	−54	−12
	Right	10.08	30	−40	−24
Lingual gyrus	Right	10.01	12	−8	−10
Lateral occipital gyrus	Left	8.19	−28	−88	12
	Right	8.35	26	−86	20
Cerebellum	Left	8.34	−34	−54	−50
	Right	10.17	30	−48	−32
Perceptual: younger > older					
Calcarine cortex	Right	4.14	12	−82	2
Perceptual: older > younger					
Precentral gyrus	Right	4.34	40	−12	64
Occipital ole	Left	4.60	−20	−100	22

CTX = cortex; FFG = fusiform gyrus; IFG = inferior frontal gyrus

The perceptual control condition elicited activation in the motor cortex and the cerebellum, which is consistent with our right-handed motor response, and also in the occipital cortex, which is consistent with the visual nature of the stimulus presentation. Age group differences during the control task are also of interest, as some groups have reported greater engagement of default-mode network regions for older adults, even while performing a task (e.g., Meinzer et al., 2012). Here, we found that older adults elicited greater activation than younger adults during the perceptual control task in the right precentral gyrus, which extended into the right postcentral and cingulate gyri and occipital pole. Younger adults elicited greater activation than older adults during the perceptual control task in the bilateral calcarine cortex.

#### Comparisons between phonological and semantic conditions

**Fig. 4:**
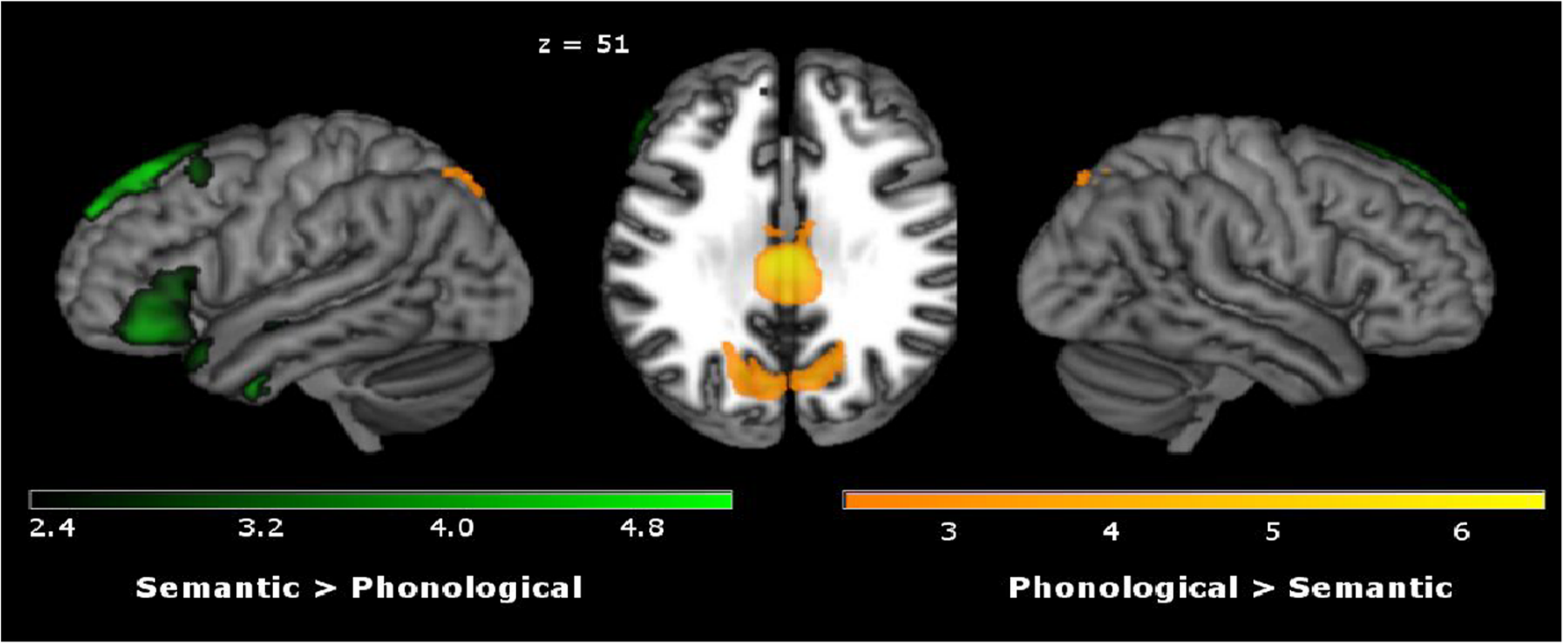
Overview of the regions that comprise the main effect of condition. Regions in which the semantic condition elicited greater activation than the phonological condition are shown in green, and regions in which the phonological condition elicited greater activation than the semantic condition are shown in orange. Colored regions represent areas where significant differences were found, *p* < .01, corrected. (Color figure online)

Collapsing across age groups, the ANOVA yielded a significant main effect of condition. The phonological condition elicited greater activation than the semantic condition in the bilateral precuneus and bilateral posterior cingulate cortex. Semantic trials elicited greater activation than phonological trials in the left hemisphere regions, including the dorsal medial prefrontal cortex (DMPFC), inferior frontal gyrus (IFG), anterior middle temporal gyrus (MTG), and fusiform gyrus (see Fig. 4). These results are summarized in Table 3.

**Table 3:** Differences between conditions

Region	Hemisphere	Voxels	Max *Z*		Peak (MNI)		
			Phonological	Semantic	*X*	*Y*	*Z*
Semantic > phonological							
SFG, frontal pole	Left	667	5.36	7.08	−8	54	34
SFG, MFG	Left	154	2.78	4.92	−24	20	56
IFG, frontal pole	Left	1,542	9.05	10.52	−46	40	−12
Temporal pole	Lef	98	1.78	2.98	−48	16	−24
MTG	Left	73	1.67	3.88	52	−8	−18
Temporal fusiform cortex	Left	187	3.42	4.77	−42	−4	−38
Phonological > semantic							
PCC	Bilateral	735	5.76	4.41	0	−20	28
Precuneus, cuneus	Left	704	9.35	7.65	−12	−72	−4
Precuneus, cuneus	Right	665	8.53	6.91	10	−74	38

IFG = inferior frontal gyrus; MFG = middle frontal gyrus; MTG = middle temporal gyrus; PCC = posterior cingulate cortex; SFG = superior frontal gyrus

#### Comparisons between older and younger adults

**Fig. 5:**
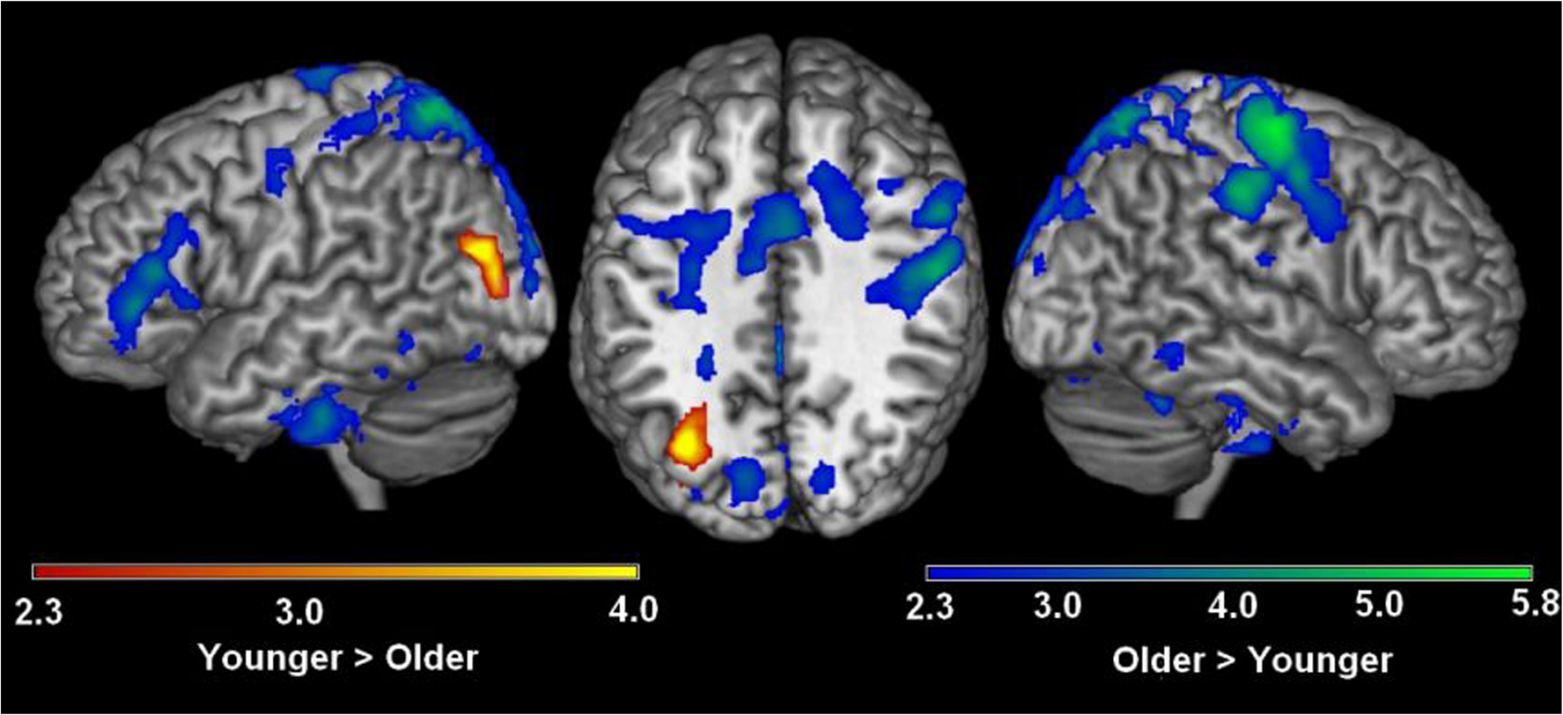
Overview of the regions that comprise the main effect of age. Regions in which younger adults elicited greater activation than older adults are shown in red, and regions in which older adults elicited greater activation than younger adults are shown in blue, *p* < .01, corrected. (Color figure online)

Collapsing across conditions, the ANOVA yielded a significant main effect of age group (see Fig. 5, Table 4). Younger adults elicited greater activation than older adults only in the occipital cortex. In contrast, older adults elicited greater activation than younger adults in many regions throughout the brain, including the left IFG, bilateral precentral and postcentral gyri, bilateral supramarginal gyri, left anterior cingulate cortex, bilateral precuneus, and bilateral inferior temporal cortices. The interaction of condition and age group was not significant in the whole-brain analysis.

**Table 4:** Age effects

Region	Hemisphere	Voxels	Max *Z*		Peak (MNI)		
			Younger	Older	*X*	*Y*	*Z*
Older > younger							
IFG	Left	1,243	13.45	13.51	−42	40	0
Precentral gyrus	Left	48	3.30	5.50	−14	−16	64
Precentral gyrus	Right	3,622	8.18	12.28	40	−12	64
Pre/postcentral gyri	Left	986	8.39	11.98	−30	−24	46
Postcentral gyrus	Left	253	6.07	8.46	−46	−40	64
Postcentral gyrus	Right	26	1.06	3.22	52	−18	18
Supramarginal gyrus	Right	785	4.17	6.57	54	−24	44
Supramarginal gyrus	Lef	103	9.18	11.34	−24	−52	34
Anterior cingulate	Left	525	5.97	10.15	−2	−8	44
ITG	Right	173	11.34	11.59	48	−46	−8
Temporal fusiform	Left	2,055	16.94	17.22	−36	−48	−12
Temporal fusiform	Right	681	16.45	17.21	38	−30	−24
Retrosplenial cortex	Bilateral	843	5.52	7.99	0	−40	14
Precuneus, SPL	Left	2,081	7.47	11.22	−4	−54	62
Precuneus	Right	1,500	6.53	9.74	8	−60	60
SPL	Right	165	4.25	7.00	32	−44	68
Occipital cortex	Left	2,149	15.89	16.73	−14	−90	2
Occipital cortex	Right	1,408	16.54	17.35	28	−76	−6
Occipital pole	Left	622	11.71	15.07	−24	−96	22
Occipital pole	Right	592	9.38	14.21	18	−90	38
Cerebellum	Right	275	15.57	16.40	34	−52	−28
Cerebellum	Right	460	10.38	10.47	16	−74	−28
Thalamus	Left	186	2.83	4.28	−4	4	−6
Thalamus	Right	223	3.83	5.44	6	2	−6
Brainstem	Bilateral	1,652	5.30	7.01	2	−30	−20
Younger > older							
Occipital cortex	Left	380	14.73	14.48	−32	−78	20

IFG = inferior frontal gyrus; ITG = inferior temporal gyrus; SPL = superior parietal lobule

#### FMRI activation-behavior relations

To investigate the relationships between activation and behavior, we first conducted a mixed-effects model with a random effect of participant and fixed effects of task and fMRI activation.^2^ We found significant main effects of task (β = −171.85, *SE* = 28.80, *z* = −5.76, *p* < .001) and fMRI activation (β = 4.77, *SE* = 1.99, *z* = 2.39, *p* < .05). To further investigate the significant effects of condition and how these differed across age groups, we conducted regressions in which age group, fMRI activation, and the interaction of age group and fMRI activation were independent variables (predictors), and efficiency (i.e., RT/accuracy) was the outcome variable. For the phonological condition, the overall model was significant, *F*(3, 36) = 5.18, *p* < .005, *R*^2^ = 0.31, although no individual predictor was.

**Fig. 6:**
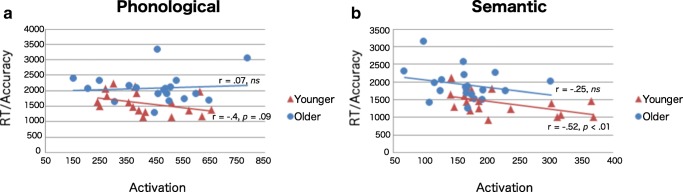
Scatterplots of the relationship between our behavioral measure of RT/accuracy and parameter estimates of the fMRI activation from the main effect of condition. **a** For the phonological condition, a significant interaction was demonstrated by a marginally significant negative correlation for the younger adults. **b** For the semantic condition, there was a significant negative correlation between RT/accuracy and fMRI activation for the overall group

For the semantic condition, the overall model was significant, *F*(1, 36) = 7.42, *p* < .001, *R*^2^ = 0.40, and fMRI activation to the semantic condition was also a significant predictor of the efficiency of semantic decisions, *F*(1, 36) = 2.13, *p* < .05. Collapsed across both groups, higher efficiency was associated with increased activation on the semantic trials (*r* = −.50, *p* < .005), and these patterns of activation were found within core language regions within the left hemisphere. Finally, we examined the relations between efficiency combined across conditions and the patterns of activation associated with the main effect of age group (older > younger). The overall model was significant, *F*(1, 36) = 5.72, *p* < .005, *R*^2^ = .34, although no individual predictor was. We should note that a Wald test revealed that the parameter estimates for the fMRI activation variable were not statistically different between the phonological and semantic models, and only trending toward significance comparing the semantic and age models (*p* = .07; fMRI parameter estimates: phonological = −11.811, semantic = −16.300, age = −0.7369) (Fig. 6).

#### Comparisons with our previous experiment

**Fig. 7:**
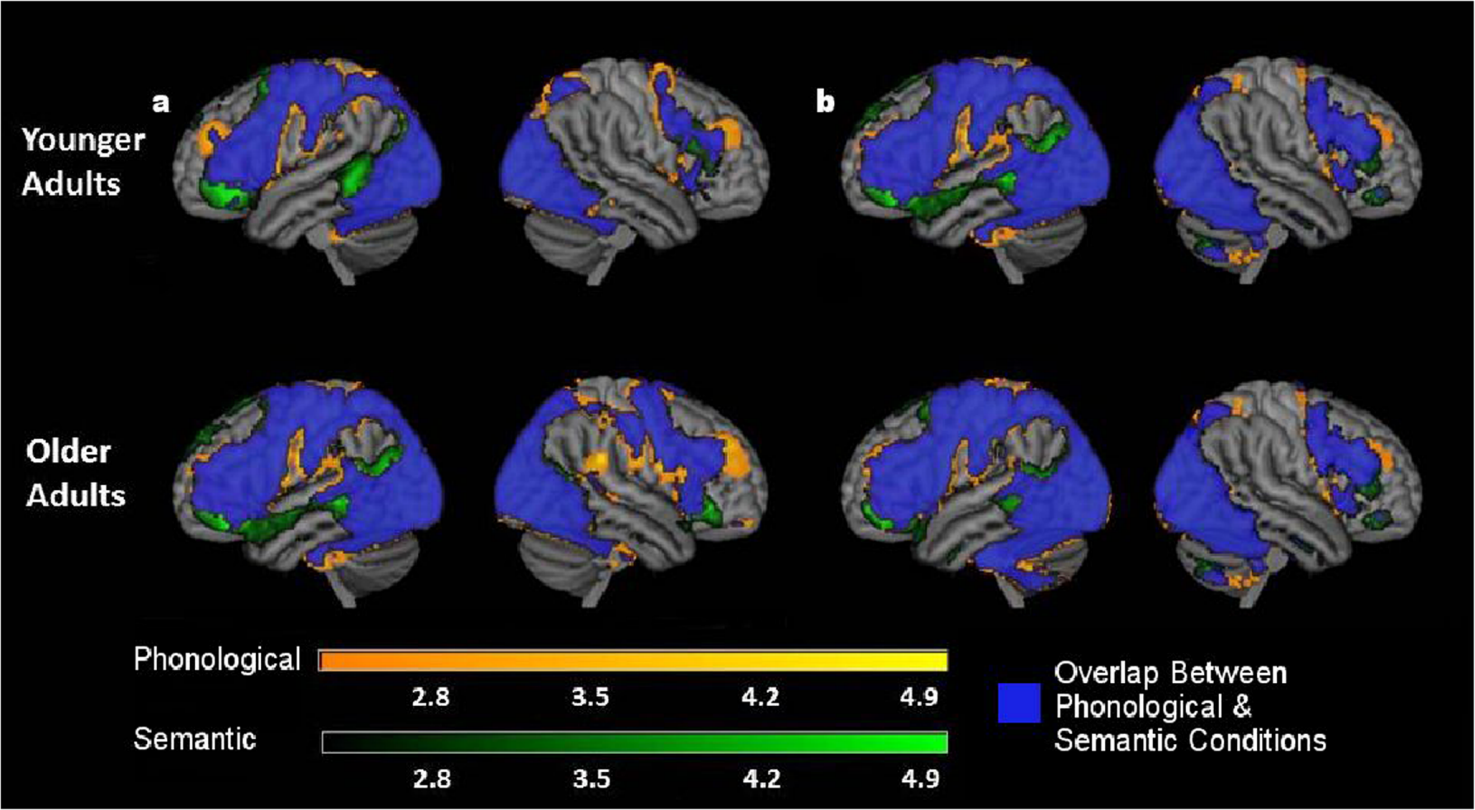
Comparison of results from Diaz et al. (2014) with the current study. Overview of the regions that were significantly activated to the phonological (orange) and semantic (green) conditions, by experiment and age group, *p* < .01, corrected. **a** Results from Diaz et al. (2014). **b** Results from the present study. Areas of overlap, where both phonological and semantic conditions elicited significant activation are shown in blue. (Color figure online)

As we did with our behavioral results, to further assess the influence of additional, task-irrelevant information on behavioral performance, we conducted a comparison between the fMRI results from the present study and those from our earlier experiment (Diaz et al., 2014). As can be seen from Fig. 7, the patterns of activation are overwhelmingly similar. The comparison of primary interest was whether older adults’ patterns of brain activation differed across the studies. For the semantic condition, there were no regions where older adults from the present study (with additional, task-irrelevant information) elicited more activation than older adults from our earlier study. For the phonological condition, older adults from the present study elicited greater activation than older adults from our earlier study in the bilateral caudate and a small region in the right posterior cingulate. Interestingly, a similar pattern in different regions was also observed for the younger adults. There were no differences in the semantic condition across experiments; however, participants from the present study elicited significantly more activation than did participants from Diaz et al. (2014) in the left middle frontal gyrus, left cingulate, bilateral posterior middle temporal gyrus, left supramarginal gyrus, left precuneus, and right lateral occipital cortex (see Table S2 for coordinates in the Supplemental Materials). Thus, when additional, task-irrelevant information was presented during phonological judgments, older adults engaged domain-general regions, largely outside of the core left hemisphere language network, whereas younger adults engaged a combination of both language-relevant regions (i.e., left MTG, SMG) as well as domain-general regions (e.g., left cingulate, precuneus, occipital regions).

## Discussion

The present experiment examined the neural and behavioral bases of phonological and semantic processing in older and younger adults. Specifically, we were interested in whether the presence of additional phonological and semantic information would differentially affect processing for younger and older adults. Theoretical perspectives vary on the mechanism underlying age-related cognitive decline, with some accounts positing that older adults experience declines in inhibition (Hasher & Zacks, 1988; Lustig, Hasher, & Zacks, 2007), while others suggest that declines are due to age-related differences in transmission of information (Burke et al., 1991), and indeed these two perspectives are not mutually exclusive. In the present experiment, participants made phonological and semantic decisions about photographs in the presence of additional phonologically or semantically related material. Behaviorally, we found a main effect of age group, in which older adults responded less efficiently than younger adults did, and a main effect of condition, in which phonological trials were responded to less efficiently than semantic trials. Because older and younger adults responded similarly to semantic and phonological trials (i.e., no significant interaction), these results suggest that the presence of additional material did not differentially impair older adults’ performance. Moreover, comparisons with our first study of semantic and phonological processing in younger and older adults, where we did not include additional semantic or phonological information, revealed only small differences for the phonological condition. There were no behavioral differences in RT, or RT/ACC, although older adults from the present study performed more accurately on phonological trials compared with older adults from Diaz et al. (2014). These between-experiment comparisons also showed that older adults from the current study engaged the posterior cingulate to a greater extent during the phonological condition. These results suggest that the additional rhyme-based phonological information in the present study may have led to increased monitoring, which ultimately increased older adults’ accuracies.

The lack of widespread differences between the two experiments also suggests that the additional information was not particularly distracting to older adults. Indeed, work by our group and others using picture-word interference designs suggest that additional semantic information, in particular, should have been disruptive to performance (Abel et al., 2009; de Zubicaray, Wilson, McMahon, & Muthiah, 2001; Rizio et al., 2017). It is possible that because the semantic task did not explicitly require production, this information was less disruptive. Also, there may have been different inhibitory demands between the semantic and phonological conditions as well as between the match and nonmatch trials. Despite these differences, it is clear that the overall effect of the additional information did not lead to stark differences in behavior or functional activation.

Considering patterns of brain activation associated with phonological and semantic processing, all language conditions engaged typical language regions including inferior frontal gyrus and temporal cortex, with greater activation in the left hemisphere. Directly comparing semantic and phonological conditions, semantic trials elicited greater activation than phonological trials in well-established regions that have been previously linked to semantic processing, including the left anterior temporal lobe, middle temporal gyrus, and dorsal medial prefrontal cortex. In contrast, phonological trials elicited greater activation than did semantic trials in the bilateral precuneus and posterior cingulate. While these are not typical language regions, the cingulate is involved in task control, and has been linked to language control and monitoring during language production (Abutalebi et al., 2008; Piai, Roelofs, Acheson, & Takashima, 2013b). The precuneus has been shown to be involved in imagery and in both auditory and visual retrieval (Fletcher et al., 1995; Huijbers, Pennartz, Rubin, & Daselaar, 2011; Kosslyn, 2003). Recruitment of these domain-general regions during the phonological condition may reflect heightened competition or attentional demands that is consistent with our behavioral results, which indicated that performance was more accurate, but overall less efficient during the phonological condition.

In looking at differences across age groups, younger adults elicited greater activation than older adults did in the left occipital cortex, which has been implicated in perceptual and conceptual aspects of picture naming (Indefrey, 2011; Indefrey & Levelt, 2004). This specific finding is consistent with what others have reported as a posterior to anterior age-related shift in activation, in which older adults engage posterior regions less than younger adults do (Davis, Dennis, Daselaar, Fleck, & Cabeza, 2008). Moreover, older adults elicited greater activation than younger adults in anterior regions, including the left inferior frontal gyrus, left anterior cingulate cortex, and bilateral precentral and postcentral gyrus, as well as in other posterior regions, such as the bilateral supramarginal gyri and precuneus. While some of these regions have explicit roles in language production (e.g., premotor cortex in articulatory planning, supramarginal gyrus in phonological code retrieval and sensory-motor transformations; Hickok & Poeppel, 2004; Indefrey, 2011), many of these regions reflect executive aspects, such as the anterior cingulate’s role in inhibition and conflict monitoring (Abutalebi & Green, 2007; Roelofs, 2008). In addition to the overall increased reliance on domain-general regions that was seen for all adults during the phonological condition, these results also suggest that both tasks may have been more attentionally demanding for older adults. Consistent with Diaz et al. (2014), we observed increased recruitment of posterior cingulate during the phonological task and increased recruitment of domain-general regions, including the left middle frontal gyrus, right inferior frontal gyrus, and posterior cingulate for older adults. Our findings are also broadly consistent with other work that has looked at competition effects and found that older adults have increased recruitment of domain-general regions such as the left middle frontal gyrus and bilateral posterior cingulate (Rizio et al., 2017), as well as increased recruitment of frontal regions that may be involved in executive aspects of language, such as left inferior frontal gyrus (Zhuang et al., 2016).

We were also interested in the function of these patterns of brain activation, and relating these to behavioral performance. Using regression analyses, the strongest relations were found examining patterns of semantic processing. Here, we found that across both groups, increases in functional activation in language-related regions were associated with increased processing efficiency (i.e., decreases in RT/accuracy scores). Because this effect was found for both groups and in language-related regions, this suggests that semantic ability and engagement of semantic regions is consistent across the life span. In contrast, while the overall regression models examining fMRI activation in the phonological condition and age differences in fMRI activation significantly predicted behavioral performance, none of the individual predictors were significant. However, we should be cautious in making inferences as a Wald test indicated that the fMRI activation parameters did not differ between the phonological and semantic models, which may reflect similar effects that are just under and just over traditional significance standards. A similar Wald test comparing parameter estimates from the semantic and aging models trended toward significance, suggesting that age-related increases in activation were not as closely tied to behavioral performance. One general consideration that should be noted is that our phonological condition may be reflecting more than just phonological processing per se. The phonological condition was the only condition in which participants were required to covertly name the object in order to perform the task. It is possible that participants were not covertly naming the objects during the other conditions, so conditional differences may also reflect differences in the intention to name (e.g., Strijkers & Costa, 2016) or differences between receptive and productive language more broadly.

In conclusion, our results help to shed light on cognitive and neural theories of aging. Inconsistent with the inhibition deficit theory (Hasher & Zacks, 1988), comparisons with our first experiment suggest that older adults were not more impaired by the presence of additional information. However, older adults, compared with younger adults, relied more heavily on both language-specific and domain-general regions, suggesting an increased need for monitoring and selection. Overall, performance and brain activation across the two experiments were similar (see Fig. 7). The differences that were found showed higher accuracy during the phonological condition when additional phonological information was present. We observed consistent engagement of brain regions during the semantic condition including the anterior temporal lobe and dorsal medial prefrontal cortex across the life span. Moreover, older adults exhibited increases in activation in nonlanguage brain regions that were unrelated to behavioral performance, suggesting an age-related decline in neural efficiency.

## Electronic supplementary material

ESM 1 (DOCX 904 kb)

## Footnotes

1 Although the phonological question involved a letter cue, correct responses required phonological retrieval of the object name because orthographic retrieval is mediated by phonology (e.g., Bonin, Peereman, & Fayol, 2001).

2 Because of the overlap in variance between the random effect of participant and age group, the model becomes rank deficient with both factors included. In the first model we retained the random effect of participant, and examined effects of age with subsequent reduced models.
