# Supplementary material for: Age-related differences in the neural bases of phonological and semantic processes in the context of task-irrelevant information
Source: Cogn Affect Behav Neurosci. 2018 Nov 28;19(4):829–44. doi: 10.3758/s13415-018-00671-2 (PMC6538491; doi:10.3758/s13415-018-00671-2)
Supplement: Supplementary file 1 — (DOCX 904 kb) [file 13415_2018_671_MOESM1_ESM.docx]

**Supplemental Materials**

Behavioral Results

*Reaction time.* Analysis of the mean untransformed RT data for correct responses indicated that there was a significant main effect of Age Group in which older adults were slower than younger adults, *F*(1, 36) = 12.14, *p* < .01. There was an additional main effect of Condition in which participants responded more quickly in the semantic condition than in the phonological condition, *F*(1,36) = 80.24, *p* < .0001. We also observed a significant main effect of Match, *F*(1,36) = 27.44, *p* < .0001, and a significant Condition x Match interaction, *F*(1,36) = 45.73, *p* < .0001. Overall, match responses were faster than non-match responses, and this difference was significant in the phonological condition, *F*(1,36) = 46.04, *p* < .0001, but not in the semantic condition. Thus, with the exception of the Age Group x Condition X Match interaction, the untransformed RT and RT/accuracy results largely mirrored each other.

Effects of Match – Non-Match decisions

*RT/ACC.* Analysis of RT/ACC indicated a significant main effect of Match, F(1,36) = 310.86, *p* < .005, and a significant Condition x Match interaction, F(1,36) = 88.25, *p* < .0001. In the phonological condition, performance was less efficient on non-match relative to all other conditions, and there were no significant differences in efficiency across the other conditions. Finally, there was a significant Age Group x Condition x Match interaction, F(1,36) = 6.03, *p* < .05. Both groups showed similar patterns in which performance on phonological non-match trials was less efficient than all other conditions, and this difference was larger for older adults than for younger adults.

*Accuracy.* A logistic regression of accuracy indicated that there was also a significant Condition x Match interaction, (β =-.91, SE = .36, z = -2.53, *p* < .01), in which participants were significantly more accurate on match trials in the phonological condition. There was no difference in accuracy in the non-match conditions.

Effects of the Perceptual Condition

*RT/ACC.* Including the perceptual condition in an ANOVA analysis of RT/ACC yielded identical results to the reduced ANOVA including only the main conditions of interest (phonological and semantic). An ANOVA of RT/ACC in which we included effects of Condition, Group, and Match indicated a significant main effect of Condition, F(1,36) = 106.53, *p* < .001, in which the perceptual condition was responded to significantly faster than the phonological condition (*t =* 11.84, *p* < .001) and the semantic condition (*t =* 10.40, *p* < .001). There were also significant differences between the phonological and semantic condition, in which participants responded faster to semantic trials (*t = 2.05*, *p* < .05). There was also a significant main effect of Group, F(1,36) = 75.75, *p* < .0001, in which older adults responded more slowly than younger adults. Finally, there was a significant Condition x Match interaction F(1,36) = 9.54, *p* < .001. In the phonological condition, performance was less efficient on non-match relative to all other conditions, and there were no significant differences in efficiency across the other conditions.

*Accuracy.* A mixed logistic regression was conducted on the number of response errors across perceptual, phonological, and semantic conditions to explore the effect of Condition and Age, with random effects of subject and item slopes (Jaeger, 2008). This analysis indicated that there was a significant effect of Condition in which participants responded more accurately to the perceptual condition (β = -.719, SE = .10, z = -6.63, *p* < .001).

Results: *fMRI Activation – Behavior Relations with Reaction Time (RT)*

To investigate the relationships between activation and behavior we conducted linear regressions in which Age Group, the parameter estimates of the fMRI activation, and the interaction of Age Group and fMRI activation were independent variables (predictors), and untransformed RT was the outcome variable. Similar analyses conducted with RT/accuracy as the outcome variable are described in the main manuscript.

For the phonological condition, the overall model was significant (F(1,36) = 5.97, *p* < .005, R^2^ = 0.35), and there was a significant interaction between Age Group and fMRI activation (F(1,36) = 2.13, *p* < .05). Examination of the correlations revealed that the interaction between RT and activation to the phonological condition was driven by a significant negative correlation for the younger adults (younger: *r* = -0.57, *p <* .01; older: *r* = 0.11, n.s.).

For the semantic condition, the overall model was significant (F(1,36) = 7.45, *p* < .001, R^2^ = 0.40) and fMRI activation to the semantic condition was also a significant predictor of RT (F(1,36) = 2.67, *p* < .01). The correlation indicated that there was a negative relationship between RT/accuracy and fMRI activation (*r* = -0.54, *p* < .0005). Collapsed across both groups, faster response times were associated with increased activation to the semantic trials.

Finally, we also examined the relations between RT and the patterns of activation associated with the main effect of Age. The overall model was significant (F(1,36) = 4.97, *p* < .01, R^2^ = 0.31), although no individual predictor was. A step-wise regression with the same variables indicated that there was a significant relationship between behavior and the interaction term that accounted for a similar amount of variance as the overall model (F(1,36) = 13.56, *p* < .001, R^2^ = 0.27). Examination of the correlations revealed that the interaction between RT and activation to the main effect of Age was driven by a marginally significant negative correlation for the younger adults (younger: *r* = -0.42, *p =* .07; older: *r* = -0.05, n.s.).

**Supplemental Figure 1**

Representative examples of the original and scrambled images that were used as a perceptual baseline.


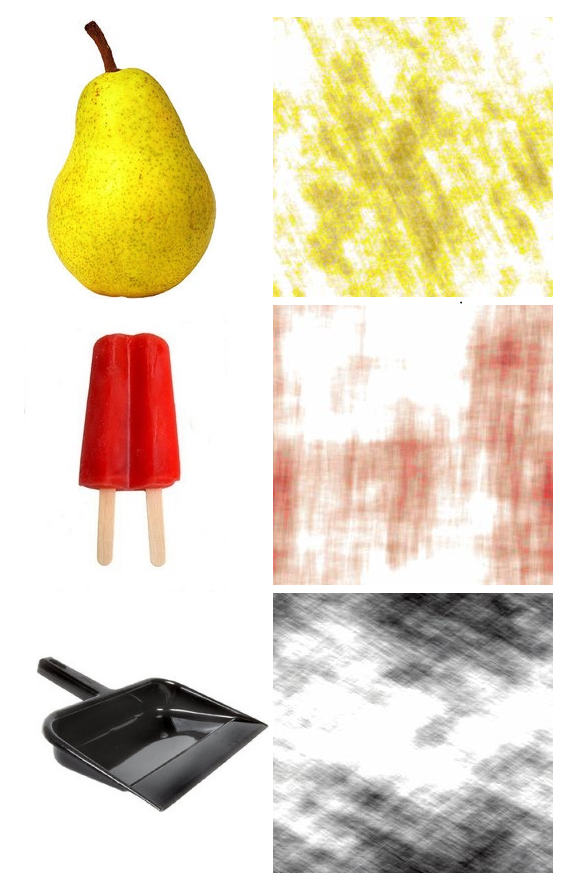


**Supplemental Table 1: Main Effect of Age with Sub-peaks**

**Max Z Peak (MNI)**

**Region Hemisphere Voxels Younger Older X Y Z**

Older > Younger

IFG left 1,243 13.45 13.51 -42 40 0

pars triangularis left -40 30 12

frontal pole left -42 40 0

precentral gyrus right 3,622 8.18 12.28 40 -12 64

precentral gyrus right 40 -12 64

SFG right 18 -4 56

temporal fusiform left 2,055 16.94 17.22 -36 -48 -12

occipitotemporal fusiform left -36 -48 -12

temporal fusiform left -38 -10 -28

precuneus, SPL left 2,081 7.47 11.22 -4 -54 62

precuneus left -4 -54 62

SPL left -22 -58 66

occipital cortex left 2,149 15.89 16.73 -14 -90 2

occipital pole left -14 -90 2

occipital fusiform left -26 -76 -10

occipital cortex right 1,408 16.54 17.35 28 -76 -6

occipital fusiform right 28 -76 -6

lingual gyrus right 28 -60 -4

**Supplemental Table 2: Experimental Differences Younger adults**

Regions in which participants from the current study elicited significantly more fMRI activation than participants in Diaz et al., 2014

**Peak (MNI)**

**Region Hemisphere Voxels Max Z X Y Z**

MFG left 1,021 4.36 -46 30 24

MTG left 365 4.05 -58 -44 2

MTG right 64 3.59 46 -48 4

Cingulate left 46 4.16 -10 -24 26

SMG left 135 3.13 -36 -48 30

Precuneus left 84 3.11 -24 -58 14

Lateral Occipital right 336 3.53 58 -64 11

MFG = middle frontal gyrus; MTG = middle temporal gyrus; SMG = supramarginal gyrus
